# Fine‐Scale Population Genomics Reveals Genetic Differentiation in the Brooding Amphipod *Cheirimedon femoratus* Across the South Shetland Islands, Antarctica

**DOI:** 10.1002/ece3.74336

**Published:** 2026-09-08

**Authors:** Euna Jo, Vahid Sepahvand, Yong‐Uk Ahn, Seunghyun Kang, Miles Lamare, Ceridwen Fraser, Jee‐Hoon Kim, Sanghee Kim

**Affiliations:** ^1^ Division of Life Sciences Korea Polar Research Institute Incheon South Korea; ^2^ Department of Marine Science University of Otago Dunedin New Zealand; ^3^ Department of Biological Sciences and Bioengineering Inha University Incheon South Korea; ^4^ Division of Ocean & Atmosphere Sciences Korea Polar Research Institute Incheon South Korea

**Keywords:** Amphipoda, biogeography, GBS, genotyping‐by‐sequencing, population structure

## Abstract

Antarctic marine ecosystems are sensitive to environmental change, and impacts on processes such as population connectivity will play a fundamental role in future population dynamics and persistence, affecting short‐term demography and long‐term evolution. We investigated the population genomics of the common benthic brooding Antarctic amphipod *Cheirimedon femoratus* (Pfeffer, 1888), using 8837 high‐quality single‐nucleotide polymorphisms (SNPs) from 87 individuals collected at 4 sites in the South Shetland Islands, separated by up to 200 km: Deception Island, King George Island, Livingston Island, and Snow Island. While Admixture, *F*_ST_, principal component analysis (PCA), and demographic (Ne) analyses revealed a generally weak population genetic structure, Livingston Island emerged as a distinct population, especially compared to King George Island. All populations showed a heterozygote deficit with positive inbreeding coefficients (*F*_IS_), particularly high in the Snow Island population (~0.55). Tajima's *D* test suggested overall neutral evolution, although slight variation was observed among sites. Despite the limited dispersal potential of this brooding species, the observed connectivity may be maintained through passive dispersal, likely via floating macroalgae or ice‐rafted debris, facilitated by prevailing regional ocean currents. This may enhance the population resilience of Antarctic benthic communities under environmental change, including regional warming and shifts in ocean circulation, compared to more isolated populations. Our findings underscore the complex interplay between passive connectivity and fine‐scale differentiation in shaping Antarctic benthic invertebrate diversity.

## Graphical abstract

Using genotyping‐by‐sequencing‐derived single‐nucleotide polymorphisms, we investigated population connectivity in the Antarctic benthic amphipod *Cheirimedon femoratus* across the South Shetland Islands. Despite the limited dispersal potential of this brooding species, populations showed substantial genetic connectivity, likely maintained through passive dispersal via ocean currents and rafting. However, the Livingston Island population exhibited localized genetic divergence, suggesting that environmental and historical factors may disrupt connectivity at fine spatial scales.

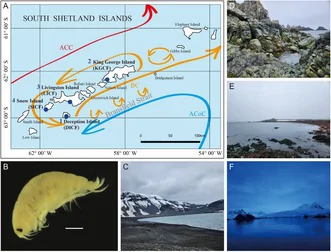

## 1 Introduction

The Southern Ocean has traditionally been considered a region of high connectivity and homogeneous populations, driven by ocean currents such as the Antarctic Circumpolar Current (ACC) and the Antarctic Coastal Current (ACoC) (Riesgo et al. 2015). However, patterns vary dramatically among species (Moon et al. 2017). For example, the sea urchin *Sterechinus neumayeri* shows broad‐scale genetic homogeneity across the Southern Ocean (Díaz et al. 2018), whereas in benthic octopod species that are closely related with similar life histories, they can exhibit contrasting spatial genetic structures at regional scales (Strugnell et al. 2017). Such variability is reflected in many Antarctic benthic species with circum‐Antarctic and eurybathic distributions (Brey et al. 1996; Clarke 1996). The ACC and Antarctic Convergence (also referred to as the polar front, PF), however, act as physical and physiological barriers that can partially isolate the Antarctic region (Barnes et al. 2006; Clarke et al. 2005; Fraser et al. 2012), with transgressions generally only observed for strong swimmers or for rafts at the very surface of the ocean (Fraser et al. 2017, 2018). For many taxa, this has resulted in limiting gene flow between Antarctic species and those from lower latitudes. Combined with biogeographical/physical isolation, glacial–interglacial cycles have contributed to a high degree of endemism and to the diversity of benthic species in the Southern Ocean (Kaiser et al. 2013; Riesgo et al. 2015). For example, during glacial maxima, small populations survive in ice‐free glacial refugia that serve as sources for recolonization after glacial periods (Allcock and Strugnell 2012; Thatje et al. 2005). Such cycles can lead to population bottlenecks and cryptic speciation that can be detected in modern population genetic structures (Allcock and Strugnell 2012; Fraser et al. 2012; Hewitt 1996). For this reason, population studies have examined the genetic connectivity of several key marine benthic fauna in the Southern Ocean (Dambach et al. 2012; Díaz et al. 2018; Dömel et al. 2015; Lau et al. 2023), including several studies on Antarctic amphipods (Riesgo et al. 2015; Thatje 2012).

Intriguing patterns have been revealed in sub‐Antarctic and Antarctic crustaceans that can shed light on the evolutionary impacts of past processes. Previous research on the genetic diversity and population connectivity in the Antarctic amphipods *Orchomenella franklini* and *Orchomene sensu lato* has suggested that genetic variation may have arisen through distinct recolonization pathways from separate refugia (Baird et al. 2011; Havermans et al. 2011). That cryptic speciation has occurred in both species, for two kelp‐dwelling sub‐Antarctic crustaceans, including the intertidal amphipod, *Parawaldeckia kidder*, long‐range movement is likely facilitated by macroalgal rafting (Nikula et al. 2010). Consequently, amphipod populations exhibit population genetic structures very similar to those of their host algae (Nikula et al. 2010). These earlier studies focused on macro‐scale connectivity and primarily used the single mitochondrial cytochrome oxidase subunit I (*COI*) gene (Havermans et al. 2011; Nikula et al. 2010), with one study additionally employing mitochondrial cytochrome b (*CytB*) and nuclear internal transcribed spacer region 2 (*ITS2*) genes (Baird et al. 2011). A subsequent study on *O. franklini* emphasized the importance of fine‐scale investigation and, using several microsatellite markers, provided evidence for ongoing speciation in two regions of East Antarctica (Baird et al. 2012).

In the present study, the genetic structure and connectivity in the ecologically important Southern Ocean amphipod, *Cheirimedon femoratus* (Pfeffer, 1888), are examined in populations inhabiting the South Shetland Islands. *Cheirimedon femoratus* has a distribution encompassing coastal Antarctica, the South Georgia area, and the sub‐Antarctic Indian Ocean, and occurs in benthic environments at depths ranging from 0 to 1500 m (De Broyer et al. 2007; Krapp et al. 2008). *Cheirimedon femoratus* lacks a free‐living larval stage and has direct development, which involves brooding a small number of embryos (< 100) for about 6 months. After this time, they are released as benthic juveniles (Bregazzi 1972).

Life‐history characteristics and population connectivity are important for population dynamics and resilience of marine taxa (Bernal‐Durán et al. 2024; Cowen and Sponaugle 2009). For most marine benthic invertebrates, the planktonic larval stage drives key population processes, including dispersal, connectivity, recruitment, geographic distributions, and stability (Sinigaglia et al. 2024). In contrast, brooding species such as *C. femoratus* will likely exhibit lower connectivity and stronger genetic structure because they lack a dispersive larval stage. For example, the Antarctic brooding benthic microbivalve *Kidderia subquadrata* shows strong population genetic structure (Levicoy et al. 2021), whereas the Antarctic phyllodocid annelid *Pterocirrus giribeti*, with planktonic larvae, exhibits panmixia and long‐distance dispersal (Leiva et al. 2018). For the brooding invertebrates, passive dispersal of juvenile and adult stages may provide an alternative mechanism for maintaining connectivity among distant populations, for example, by attaching to drifting algae or debris carried by ocean currents (Winston 2012). These contrasting expectations raise the question of how dispersal shapes population structure in *C. femoratus* . Given this uncertainty, we examined if brooding amphipods exhibit fine‐scale genetic connectivity in fragmented Antarctic habitats. We hypothesized that limited dispersal would result in significant fine‐scale genetic differentiation among islands, or alternatively, passive dispersal could maintain some degree of connectivity among populations.

**FIGURE 1:**
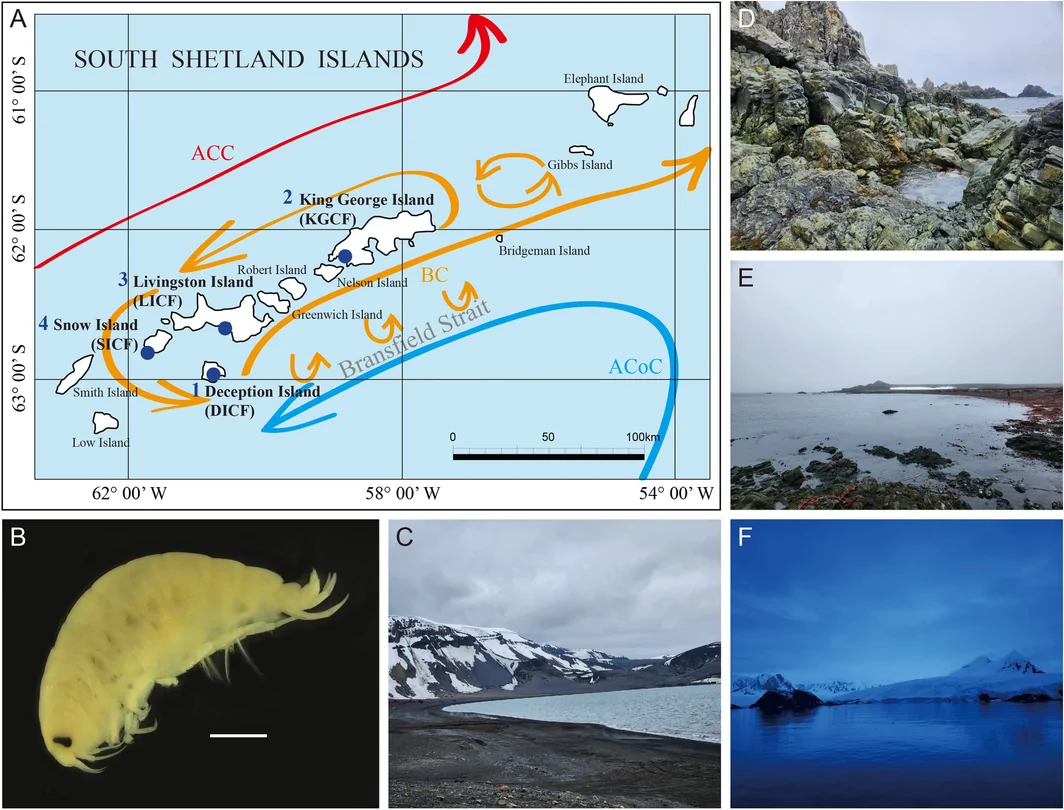
(A) The South Shetland Island group, with the location of four *Cheirimedon femoratus* population samples indicated and numbers (corresponding to Table 1 locations). Also shown are the major oceanographic currents in the region based on Sangrà et al. (2017) and Servettaz et al. (2025). The arrows show the circulation of surface waters: The Antarctic Circumpolar Current (ACC, red), the Antarctic Coastal Current (ACoC, blue), and the Bransfield Current (BC, orange). (B) Habitus of a *C. femoratus* specimen SNCF005 (scale bar: 2.0 mm). (C–F) Photographs of sampling sites: (C) Deception Island (DICF), (D) King George Island (KGCF), (E) Livingston Island (LICF), and (F) Snow Island (SICF).

To address this, we examined the genetic structure of the Antarctic amphipod *C. femoratus* across the South Shetland Islands. The South Shetland Islands, located north of the West Antarctic Peninsula, comprise 11 major islands that extend roughly linearly over 500 km between 61° S and 63° S. The region is tectonically active (Bastías et al. 2023; Dai et al. 2025) and sits in an area of relatively complex oceanography, with the islands influenced by the strong eastward flowing ACC to the north and the westward‐flowing ACoC from the Weddell Sea to the south (Sanchez et al. 2019). The Peninsula Front separates these flows, and north of this is a narrow north‐easterly flowing jet, the Bransfield Current (BC), that travels along the southern shores of the islands at rates of 0.3–0.4 m s^−1^ (Figure 1). The flow of these currents is important in biologically relevant transport processes, including population connectivity, movement of propagules, and drift algae. The habitat fragmentation and environmental heterogeneity that may exist between these islands, as well as the complex oceanography, place this region in a state of scale‐dependent population connectivity. To this end, we used a genotyping‐by‐sequencing (GBS) approach, which provides genome‐wide single‐nucleotide polymorphism (SNP) data at higher resolution than traditional genetic markers such as mitochondrial DNA or microsatellites (Wang et al. 2020). Using this approach, the main objectives of this study were to: (1) quantify the fine‐scale population genetic structure of *C. femoratus* across the South Shetland Islands; (2) determine whether the structure indicates barriers to gene flow or patterns of genetic connectivity; and (3) interpret the observed patterns of genetic structure in relation to the region's oceanographic and habitat characteristics.

## 2 Materials and Methods

### 2.1 Sampling and Study Area

A total of 96 samples of *C. femoratus* were collected from 4 locations in the South Shetland Islands: Deception Island (DICF), King George Island (KGCF), Livingston Island (LICF), and Snow Island (SICF) (Figure 1; Table 1). KGCF samples were collected manually from a tide pool in the intertidal zone using a hand net (see Figure 1D for sampling location images). The other samples were collected during the Antarctic expedition aboard the RV *Betanzos* from January 15 to 29, 2024. SICF samples were collected using a baited trap at a depth of approximately 50 m, about 1 km offshore from the vessel (Figure 1F), while DICF and LICF samples were collected by hand‐netting in the intertidal zone (Figure 1C,E). All collected samples were immediately fixed in 95% ethanol and stored at −20°C until analysis. The software packages QGIS v3.22 (http://www.qgis.org) and Quantarctica (Matsuoka et al. 2021) were used to create detailed maps of the sampling locations. A habitus photograph of specimen SNCF005 was taken using a stereomicroscope (S8 APO, Leica, Wetzlar, Germany).

**TABLE 1:** Four South Shetland Island sampling locations of *Cheirimedon femoratus*, and details of sample size and sampling data.

No.	Symbol	Name of site	Latitude	Longitude	Sampling date	No. individuals (initial/final)
1	DICF	Deception Island	62°58′5.45″ S	60°42′33.36″ W	January 23, 2024	24/24
2	KGCF	Near King Sejong Station, King George Island	62°13′12.29″ S	58°46′49.76″ W	January 1, 2024	25/23
3	LICF	Punta Hannah, Livingston Island	62°38′38.20″ S	60°37′17.06″ W	January 20, 2024	24/21
4	SICF	Snow Island	62°44′54.54″ S	61°12′32.64″ W	January 19, 2024	23/19

*Note:* Initial *N* = number of individuals collected; final *N* = number retained after excluding samples that failed library preparation, barcode demultiplexing, or quality‐control filtering.

### 2.2 GBS Library Preparation and Sequencing

We selected the GBS approach as a cost‐effective, high‐throughput method for generating genome‐wide SNP data, well suited to non‐model organisms (Vaux et al. 2023). Whole genomic DNA was extracted from subsamples of fixed *C. femoratus* using DNeasy Blood & Tissue Kit (Qiagen, Hilden, Germany). Samples were rinsed with pure water before DNA extraction, which followed the manufacturer's protocols, except that the lysis step was extended overnight with Proteinase K, and the DNA extracts were then treated with RNase A. The DNA concentration was measured using a Qubit 2.0, then stored at −20°C for later GBS library preparation. We followed a standard single‐digest GBS protocol (Elshire et al. 2011) using the restriction enzyme PstI (New England Biolabs, Ipswich, MA, USA) and T4 DNA Ligase (Thermo Fisher Scientific, Waltham, MA, USA). PCR amplification was made using 2× TOPsimple Master Mix (Enzynomics, Daejeon, South Korea), GBS PCR primers forward (5′‐AATGATACGGCGACCACCGAGATCTACACTCTTTCCCTACACGACGCTCTTCCGATC*T) and reverse (5′‐CAAGCAGAAGACGGCATACGAGATCGGTCTCGGCATTCCTGCTGAACCGCTCTTCCGATC*T), where * indicates phosphorothioation. The PCR mixture volume was prepared following the manufacturer's protocols and performed under the following conditions: 72°C for 5 min, 95°C for 2 min, 25 cycles of 95°C for 30 s, 65°C for 30 s, 72°C for 30 s, followed by a final extension step of 72°C for 5 min. PCR products were run in a 1% TAE agarose gel electrophoresis, the intensity of which was equalized using the DNA smear. Finally, PCR products were pooled in roughly equal amounts from each sample, and the pooled libraries were purified using a Nucleospin Gel & PCR Purification Kit (Macherey‐Nagel, Düren, Germany). The purified library was sent to PHYZEN Co. (Seongnam, South Korea) do for sequencing. Sequencing of the libraries was carried out on an Illumina NovaSeq 6000 system (151 bp paired‐end).

### 2.3 Bioinformatic Analysis

The quality of the raw sequence data was checked using FastQC v0.12.1 (Andrews 2010), and contaminants and adapters were removed using BBDuk v38.81 (Bushnell 2014). As no reference genome is available for *C. femoratus* , de novo assembly of RAD loci was performed using Stacks2 (Rochette et al. 2019). We optimized Stacks2 parameters (Paris et al. 2017) to maximize SNP retention. Within‐individual mismatches were set to 2 (‐M 2), and catalog mismatches were set to 3 (‐n 3); the minimum stack depth (‐m) was left at Stacks' default value of 3. Loci with heterozygosity exceeding 0.6 were excluded (‐‐max‐obs‐het 0.6).

After variant calling, the VCF file was filtered using VCFtools v0.1.15 (Danecek et al. 2011). Genotypes with a minimum depth below 3 were filtered (‐‐minDP 3), and only SNPs present in at least 80% of samples (‐‐max‐missing 0.8) were retained. Additionally, loci with a minor allele frequency (MAF) below 0.04 were removed to exclude singletons and very low‐frequency variants prone to genotyping error in GBS datasets, while retaining variants present in multiple gene copies pooled across all populations.

Genetic differentiation was assessed using multiple approaches. Firstly, variant data were imported from a VCF file using the vcfR package v1.13.0 (Knaus and Grünwald 2017) in R version 4.4.1 (R Core Team 2024). The VCF was converted to a genind object using the vcfR2genind() function from the adegenet package v2.1.10 (Jombart 2008; Jombart and Ahmed 2011). SNP variation was then assessed using principal component analysis (PCA) implemented via the glPca() function in the adegenet v2.1.10 (Jombart 2008; Jombart and Ahmed 2011). Wright's fixation index (*F*_ST_) was calculated using Arlequin v3.5.2.2 (Excoffier and Lischer 2010) and the R‐package hierfstat v0.5‐11 (Goudet 2005). The Weir and Cockerham (1984) estimator from hierfstat was reported throughout the manuscript to maintain a consistent framework for pairwise and global *F*‐statistics. Significance of pairwise *F*_ST_ values was assessed by bootstrap resampling (999 replicates), with 95% confidence intervals excluding zero used to infer statistical significance.

The sNMF algorithm (LEA v2.8.0; Frichot and François 2015) was used to infer ancestry proportions, testing *K* = 1–10 with 50 replicates per *K* value, and bar plots were generated with PopHelper v2.3.0 (Francis 2017). The optimal *K* was selected based on minimum cross‐entropy across replicates (Figure S1). A neighbor‐joining (NJ) phylogenetic tree was constructed from Prevosti's genetic distance matrix computed across the filtered genome‐wide SNPs using the bitwise.dist() function in poppr v2.9.6 (Kamvar et al. 2014), midpoint‐rooted using phangorn v2.11, and bootstrap support assessed from 100 replicates.

Hierarchical partitioning of genetic variance was assessed using the Weir and Cockerham (1984) estimator implemented in the varcomp.glob() function of the hierfstat R package v0.5‐11 (Goudet 2005). Variance was decomposed across three hierarchical levels: within individuals, among individuals within populations, and among populations, from which the corresponding *F*‐statistics (*F*_ST_, *F*_IS_, and *F*_IT_) were derived. Significance of the among‐population variance component was assessed by permutation, with population labels randomly reassigned among individuals 999 times, and the proportion of permuted *F*_ST_ values equal to or exceeding the observed value was used to calculate a *p*‐value. To evaluate genetic diversity within populations, observed (Ho) and expected (He) heterozygosity, and the inbreeding coefficient (*F*_IS_) were calculated using hierfstat package v0.5‐11 (Goudet 2005). Mean values were calculated for each population, and the results were visualized using ggplot2 v3.5.0 (Wickham and Sievert 2009) after reshaping the data with reshape2 v1.4.4 (Wickham 2007). Nucleotide diversity (*π*) was calculated for each population using VCFtools v0.1.15 (Danecek et al. 2011) in 10 kb windows with a 5 kb step size. Differences in *π* among populations were tested using a Kruskal–Wallis rank‐sum test followed by pairwise Wilcoxon rank‐sum tests with Bonferroni correction.

Tajima's *D* (Tajima 1989) was calculated for each population using VCFtools v0.1.15 (Danecek et al. 2011) in 10 kb sliding windows with a 5 kb step size, excluding windows with missing or invalid entries. For each population, the median, mean, and proportion of windows with significantly non‐zero *D* (*p* < 0.05, assessed by normal approximation: *p* = 2 × *Φ* (−|*D*|)) were computed. Among‐population differences in Tajima's *D* distributions were tested using a Kruskal–Wallis rank‐sum test, followed by pairwise Wilcoxon rank‐sum tests with Bonferroni correction.

A folded site frequency spectrum (SFS) was computed from MAFs estimated from allele count matrices; only loci with valid genotypes and sufficient SNP representation were retained. Tajima's *D* and the folded SFS were computed on the same MAF‐filtered dataset (MAF > 0.04) described above. It should be noted that any MAF filtering removes rare variants, which introduces a slight upward bias in Tajima's *D* estimates and truncates the left tail of the folded SFS. Tajima's *D* values close to zero should therefore be interpreted conservatively. Critically, since the filter was applied uniformly to the pooled dataset prior to any population‐level analysis, the observed among‐population differences in Tajima's *D* are unlikely to be attributable to this bias.

To investigate potential gene flow among amphipod populations, we used TreeMix v1.12 (Pickrell and Pritchard 2012), which models allele‐frequency covariance to reconstruct population trees and infer migration events. A TreeMix‐compatible input file was generated from the filtered VCF using the populations program from Stacks v2.52 (Rochette et al. 2019) with the ‐‐treemix option and a population map file listing individuals and their population assignments. TreeMix models with migration edges from 1 to 10 (‐m 1 to ‐m 10) were tested. To account for linkage disequilibrium (LD), SNPs were grouped into blocks of 1000 (‐k 1000). For each migration‐edge model (*m* = 1–10), TreeMix was run with a single block‐bootstrap resampling of the allele‐frequency covariance matrix (‐bootstrap). Model evaluation was performed by extracting log‐likelihood scores from TreeMix log files (treemix_[m]_log) and plotting them against the number of migration edges. Model selection was based on changes in log‐likelihood across migration‐edge models, with the most parsimonious model selected when additional migration edges did not improve model fit. To estimate relative gene flow among populations, we calculated the effective number of migrants per generation (Nem) using Wright's (1951) island model approximation: Nem = (1 − *F*_ST_)/(4*F*_ST_). Formal directional migration methods (e.g., BayesAss, divMigrate) were not applied, as they require higher *F*_ST_ values than were observed here for reliable estimation (Faubet et al. 2007; Meirmans 2014) or were not readily available; Nem was instead used as an indirect, non‐directional proxy for gene flow. The resulting tree was visualized using the plot_tree() function in R.

Contemporary effective population size (Ne) was estimated for each population using the LD method (Hill 1981; Waples 2006). This approach infers Ne from the expected relationship between Ne and the magnitude of gametic disequilibrium among pairs of unlinked loci, whereby smaller populations generate stronger LD through genetic drift. Because LD‐based Ne estimation is particularly sensitive to missing data, a stricter individual‐level missingness filter (‐‐mind 0.20) was applied prior to Ne estimation than for other analyses, reducing the dataset from 87 to 76 individuals (DICF = 22, KGCF = 19, LICF = 16, SICF = 19; Table S4). Per‐population PED/MAP files were generated from the filtered SNP dataset using PLINK v1.9 (Chang et al. 2015), retaining only SNPs polymorphic within each population (MAF > 0.01). Pairwise squared correlations (*r* ^2^) between allele frequencies were calculated across a random subsample of 2000 SNPs per population (random seed = 42) to reduce computational burden while retaining sufficient power for estimation. The Waples (2006) bias correction was applied to account for the upward bias in *r* ^2^ introduced by finite sample size: *r* ^2^_drift = mean (*r* ^2^) − 1/*n* − 3.19/*n* ^2^, where *n* is the number of sampled individuals. Contemporary Ne was then estimated as: Ne = 1/(3 × *r* ^2^_drift). SNP pairs with fewer than eight non‐missing genotype observations or zero within‐pair variance were excluded prior to calculation. Where *r* ^2^_drift ≤ 0, Ne was recorded as infinite, indicating that no drift signal was detectable above the sampling noise threshold for that population–threshold combination.

Ninety‐five percent confidence intervals were derived by block jackknife resampling of SNP pairs over 200 blocks. To assess sensitivity to rare‐allele frequency, analyses were performed at three minimum allele‐frequency thresholds: Pcrit = 0.05, 0.02, and 0.01. The Pcrit = 0.05 estimate was used as the primary value, as lower thresholds produced unstable estimates in populations with small sample sizes, consistent with the known sensitivity of LD‐based Ne estimation to rare allele inclusion (Waples and Do 2010). Prior to Ne estimation, pairwise relatedness among all sampled individuals was assessed using PLINK v1.9 (Chang et al. 2015; ‐‐genome). No pairs exceeded the threshold for close kinship (PI_HAT > 0.25; maximum observed PI_HAT = 0.18), indicating no evidence of siblings or closer relatives among sampled individuals.

To test for isolation by distance (IBD), Mantel tests were conducted between pairwise genetic distances (population‐level *F*_ST_ or individual‐level Euclidean distances) and geographic distances (in kilometers) using the mantel() function from the vegan package (Oksanen 2015) in R. Additionally, simple linear regressions were performed to assess the relationship between genetic distances and geographic distances.

Custom R scripts that integrated the data from the viridis packages (Garnier et al. 2018), ggplot2 (Wickham and Sievert 2009), and dplyr v1.1.4 (Wickham 2015) were used for all analyses and plots.

## 3 Results

### 3.1 Genomic Diversity

Of the 96 individual samples of *C. femoratus* from the 4 populations across the South Shetland Islands (DICF, KGCF, LICF, and SICF), 3 samples failed during library preparation and 6 additional samples were excluded during the quality control filtering process, leaving 87 samples for analysis. After filtering, 8837 biallelic SNPs were retained.

**FIGURE 2:**
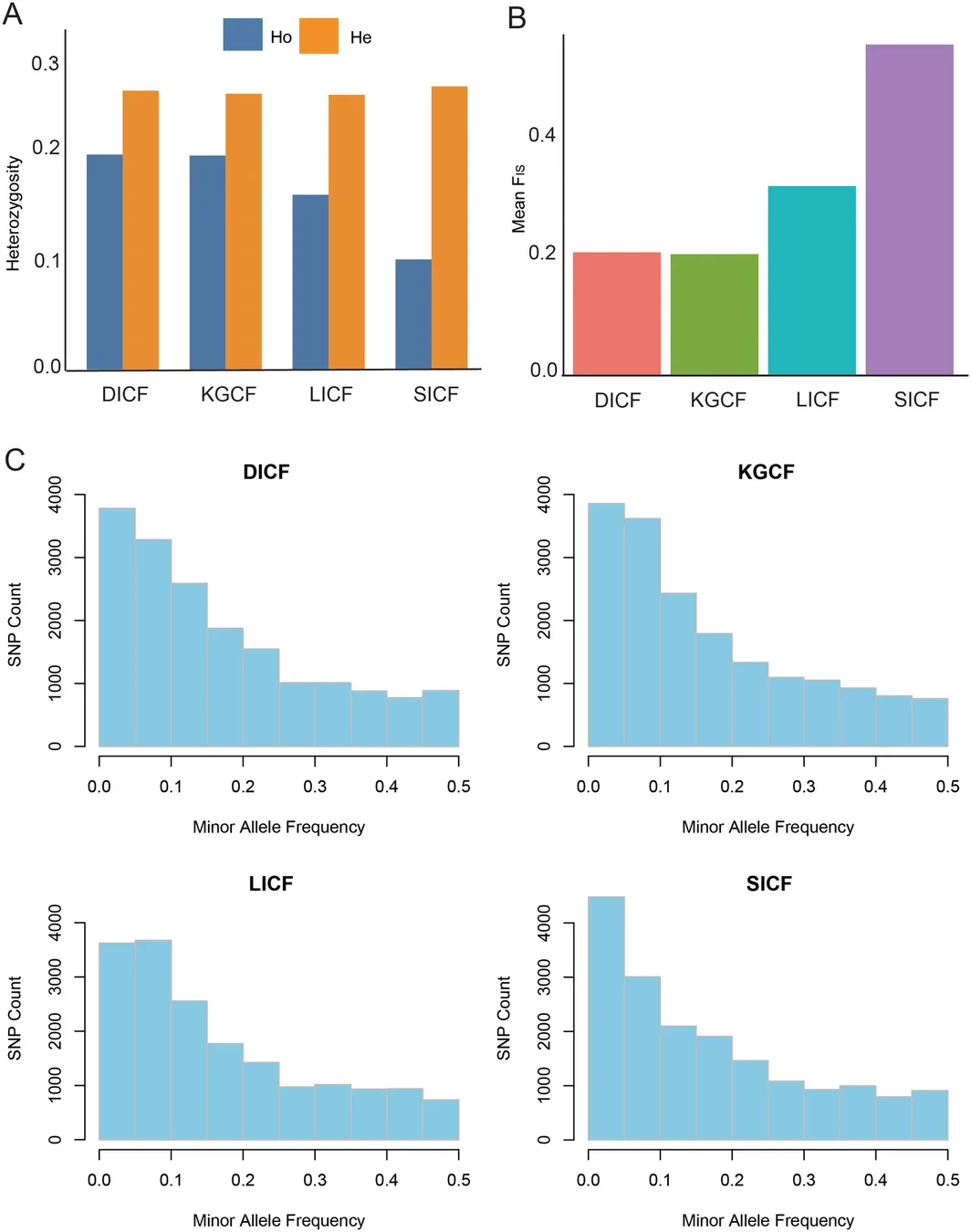
(A) Comparison of observed heterozygosity (Ho) and expected heterozygosity (He) across four *Cheirimedon femoratus* populations. (B) *F*_IS_ estimates for *C. femoratus* populations across the South Shetland Islands, reflecting deviations from the Hardy–Weinberg equilibrium. (C) Minor allele frequency (MAF) distribution for *C. femoratus* populations across the South Shetland Islands.

Across all four sites, expected heterozygosity (He) was consistently greater than observed heterozygosity (Ho), suggesting a deficit of heterozygotes (Figure 2A). The DICF and KGCF populations showed relatively low and comparable *F*_IS_ values (~0.21), and LICF showed a moderately high *F*_IS_ (~0.31). At the same time, the most westerly SICF displayed a substantially higher *F*_IS_ (~0.55) (Figure 2B).

The distribution of MAFs across the four populations revealed a consistent excess of low‐frequency variants (Figure 2C). All populations exhibited a characteristic L‐shaped spectrum, with a pronounced peak in the low‐MAF bins (0–0.05) and a gradual decline toward higher frequencies. The SICF population displayed the highest count of low‐frequency SNPs. Conversely, the central LICF population showed a slightly flatter distribution beyond MAF > 0.1, suggesting the presence of more intermediate‐frequency alleles compared with the other populations.

**FIGURE 3:**
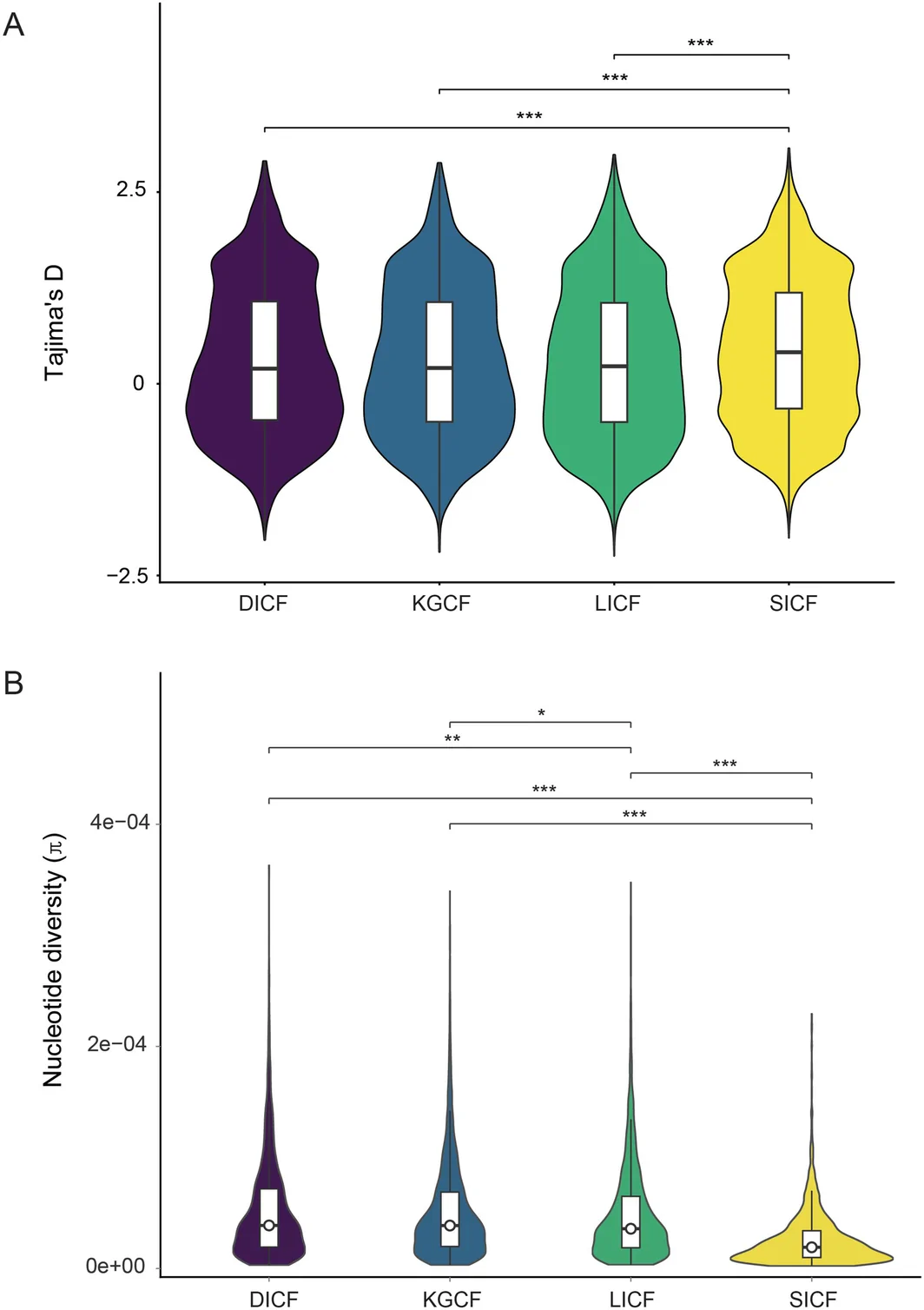
(A) Violin plots of Tajima's *D* values calculated in 10 kb windows across four *Cheirimedon femoratus* populations (DICF, KGCF, LICF, and SICF). Boxplots inside the violins show the median and interquartile range. The width of each violin represents the distribution density of Tajima's *D* estimates within each population. Kruskal–Wallis *χ* ^2^ = 55.46, df = 3, *p* < 0.0001. (B) Nucleotide diversity (*π*) across *C. femoratus* populations. Kruskal–Wallis *χ* ^2^ = 1281.4, df = 3, *p* < 0.001. Pairwise Wilcoxon tests with Bonferroni correction; *p < 0.05, **p < 0.01, ***p < 0.001.

Distributions of Tajima's *D* values varied slightly across the four sampling locations. Tajima's *D* values were close to zero across all populations, consistent with predominantly neutral evolution. Median *D* values ranged from 0.198 (DICF) to 0.411 (SICF). The proportion of genomic windows with significantly non‐zero *D* (*p* less than 0.05, normal approximation) ranged from 3.9% (KGCF) to 4.8% (SICF), approximating the expected Type I error rate under neutrality (5%) (Table S1). SICF showed a notably higher median *D* compared to the other populations, suggesting either a recent reduction in effective population size or upward bias from MAF filtering (Figure 3A). A Kruskal–Wallis rank‐sum test confirmed significant differences in Tajima's *D* distributions among populations (*χ* ^2^ = 55.462, df = 3, *p* < 0.0001). Post hoc pairwise Wilcoxon tests with Bonferroni correction revealed that SICF differed significantly from all three other populations (DICF: 0.198, KGCF: 0.206, LICF: 0.229; all *p* < 0.001), while DICF, KGCF, and LICF did not differ significantly from one another (all *p* = 1.000).

Nucleotide diversity (*π*) differed among the four *C. femoratus* populations (Kruskal–Wallis test: *χ* ^2^ = 1281.4, df = 3, *p* < 0.001) (Figure 3B). Nucleotide diversity was highest in DICF and KGCF, which did not differ significantly from each other, intermediate in LICF, which was significantly lower than both DICF (*p* < 0.01) and KGCF (*p* < 0.05), and lowest in SICF, which was significantly lower than all three other populations (*p* < 0.001 in each comparison) (Figure 3B).

### 3.2 Population Genetic Structure

**FIGURE 4:**
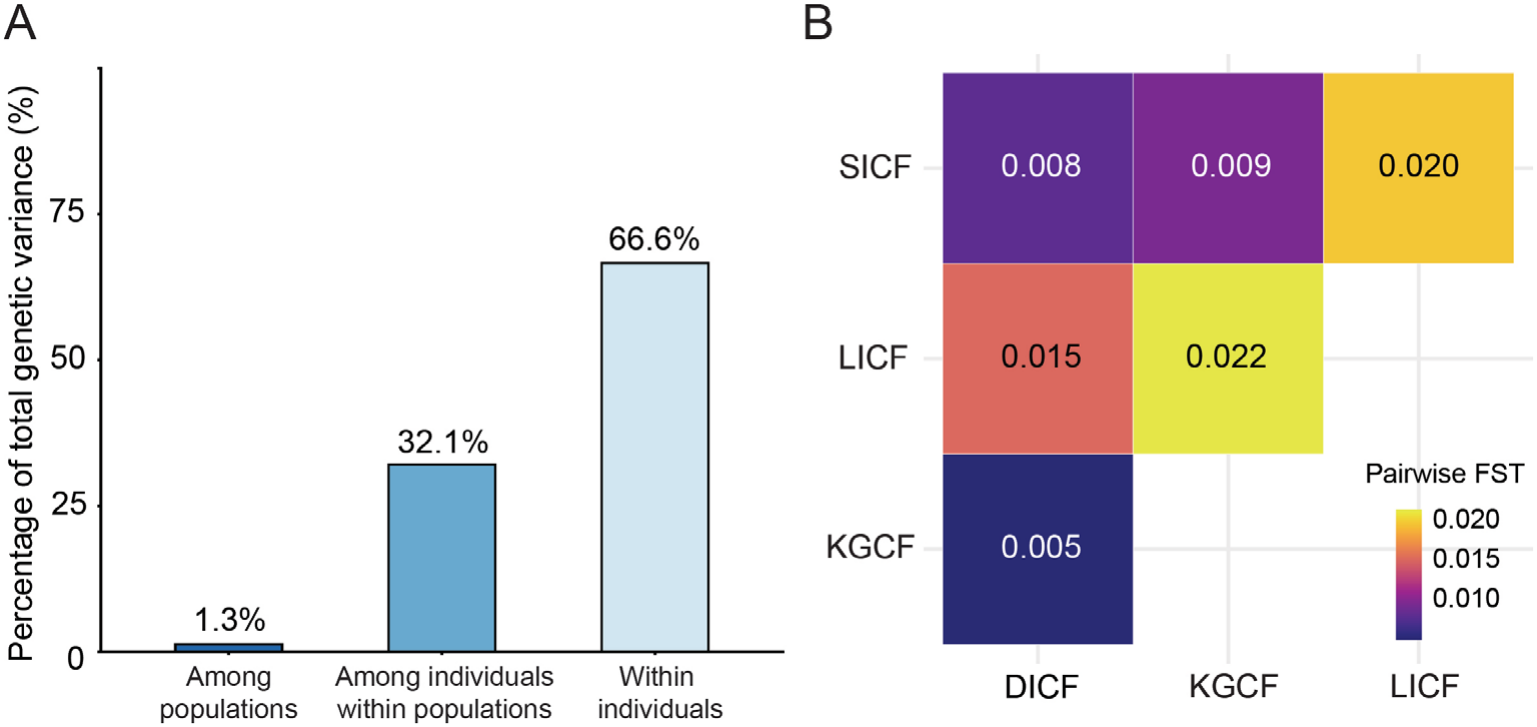
(A) Hierarchical variance decomposition showing that 66.6% of total genetic variance resides within individuals, 32.1% among individuals within populations, and 1.3% among populations (*F*_ST_ = 0.013, *p* < 0.01). (B) Heatmap of pairwise genetic differentiation (*F*_ST_) among four *Cheirimedon femoratus* populations.

Hierarchical variance decomposition from hierfstat showed that 66.6% of total genetic variance resided within individuals, 32.1% among individuals within populations, and 1.3% among populations (*F*_ST_ = 0.013, *p* < 0.01, 999 permutations; *F*_IS_ = 0.325, *F*_IT_ = 0.334) (Table S2). The variance partitioning is shown in Figure 4A. All six pairwise *F*_ST_ comparisons were statistically significant (*p* < 0.001, bootstrap 95% CIs excluding zero), ranging from 0.005 (DICF‐KGCF; 95% CI: 0.004–0.006) to 0.022 (KGCF‐LICF; 95% CI: 0.020–0.024) (Figure 4B; Table S3). Both Arlequin and hierfstat produced highly consistent pairwise *F*_ST_ estimates (maximum difference < 0.001 across all six population pairs). The highest differentiation involved LICF.

**FIGURE 5:**
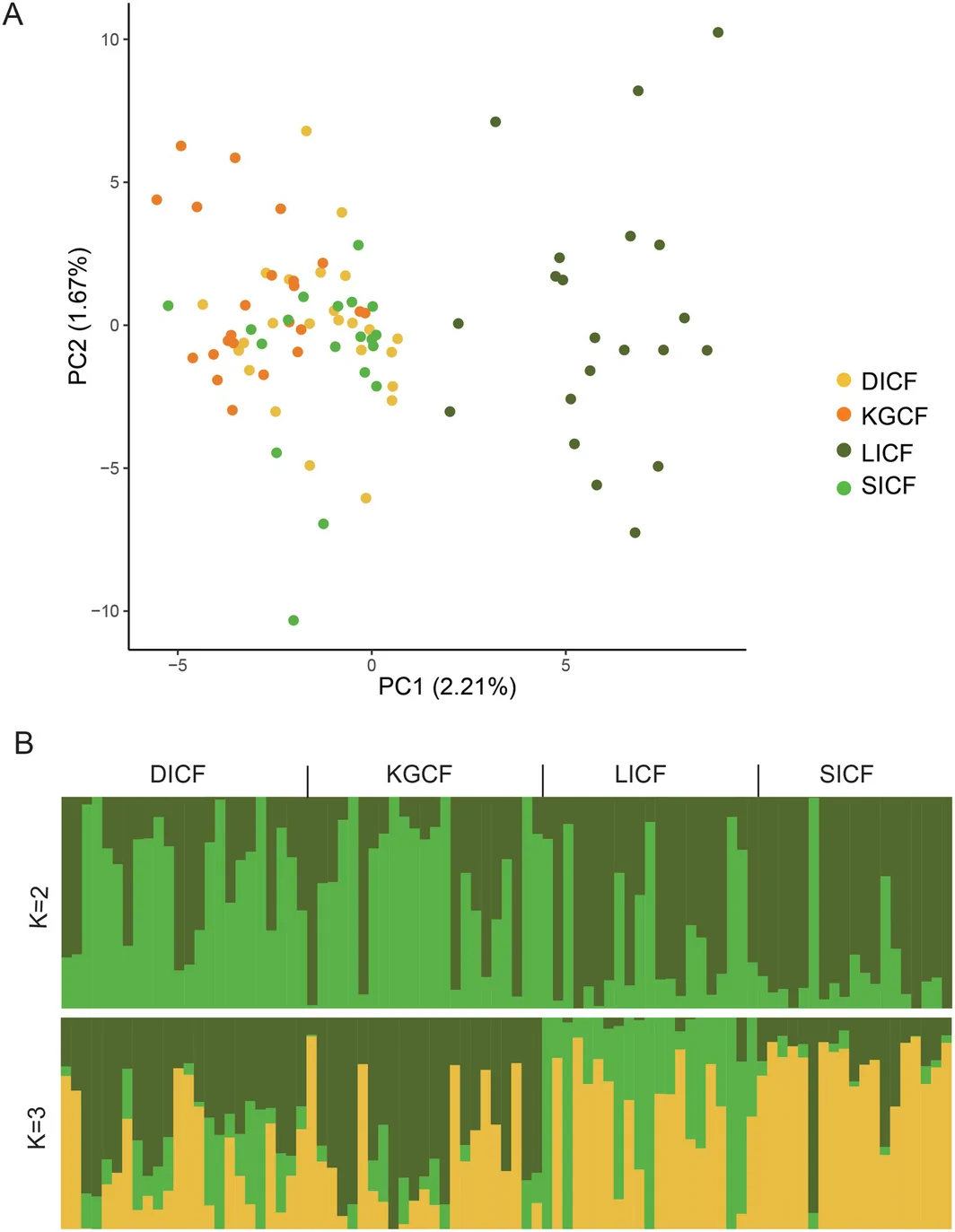
(A) Principal component analysis (PCA) plot of *Cheirimedon femoratus* individuals from the South Shetland Islands, showing genetic clustering among populations. (B) Admixture analysis of *C. femoratus* populations across the South Shetland Islands at *K* = 2 and *K* = 3, showing individual ancestry proportions.

Principal components analysis revealed that the first two principal components (PCs) explained only ~4% of the total genetic variation across all individuals (PC1: 2.21%, PC2: 1.67%; Figure 5A). Nevertheless, the PCA plot revealed a distinct clustering pattern: the LICF population showed partial separation from the other populations, whereas individuals from KGCF, DICF, and SICF formed a single overlapping cluster, suggesting greater genetic similarity among these three groups. The sNMF analysis identified *K* = 2 as the best‐supported number of ancestral populations based on cross‐entropy minimization (Figure S1). Cross‐entropy values at *K* = 2 were tightly clustered across replicates (mean = 0.681, SD = 0.0047), indicating consistent convergence among independent runs. Although *K* = 2 had the lowest cross‐entropy, *K* = 3 revealed a more pronounced distinction of the LICF group from the others. These results are consistent with weak but detectable population structure. We present *K* = 2 as the best‐supported result and *K* = 3 as an exploratory result providing additional biological insight (see Section 4; Figure 5B).

The NJ tree is consistent with the PCA and admixture results: no population formed a fully exclusive monophyletic clade (Figure S2). LICF individuals showed the strongest tendency toward clustering, although individuals from different populations remained interspersed across the tree. Bootstrap support for internal nodes was generally low (< 70%).

### 3.3 Gene Flow Among Populations

**FIGURE 6:**
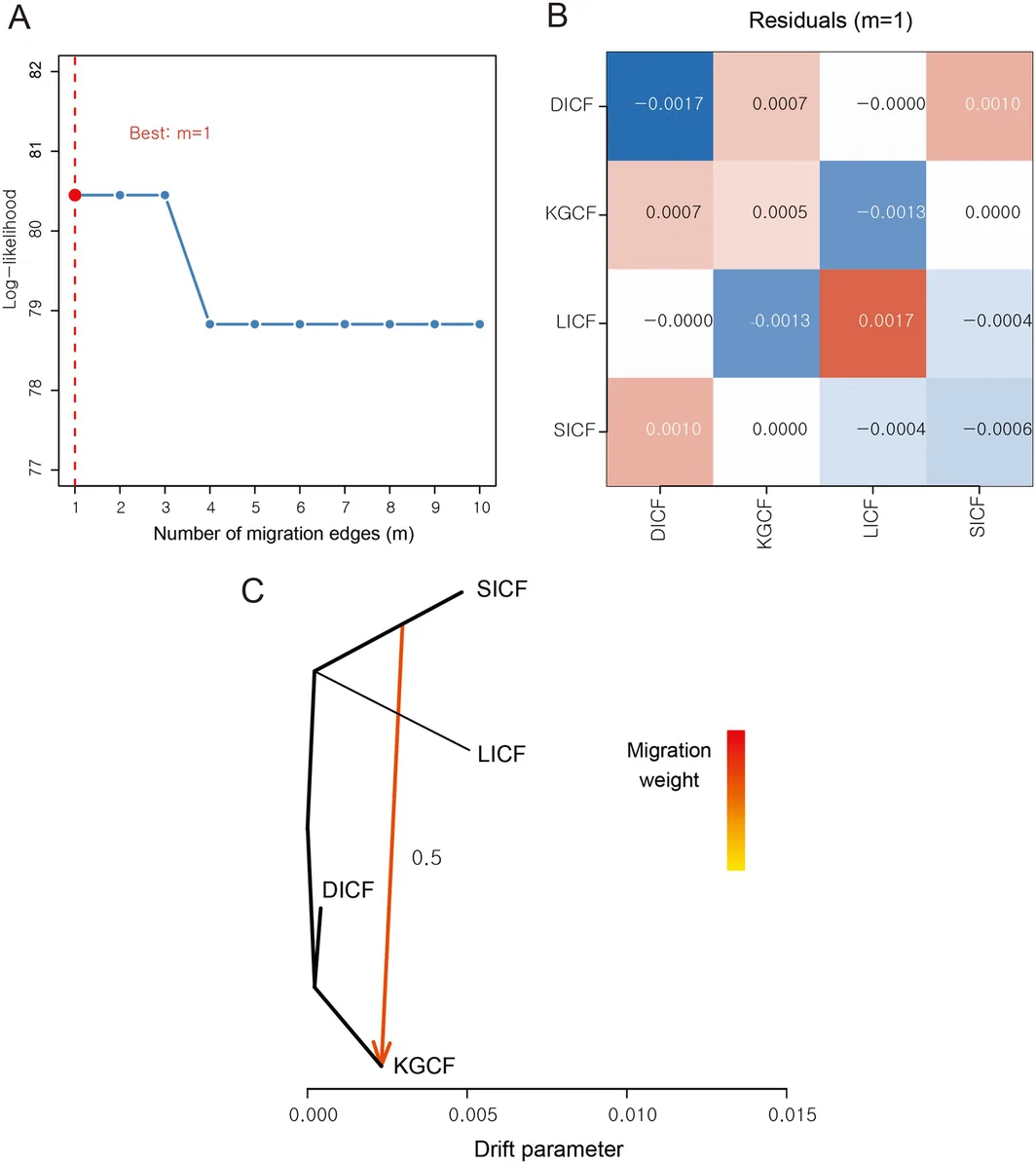
(A) TreeMix log‐likelihood scores across migration edges (*m* = 1–10), confirming *m* = 1 as the best‐supported model. (B) Residual fit matrix for the *m* = 1 model (maximum residual = 0.0017, MSE = 0.000105). (C) TreeMix population tree with *m* = 1 migration edge, showing two main clusters (DICF + KGCF; LICF + SICF) and a single migration event from the LICF/SICF ancestral lineage toward KGCF (migration weight = 0.336), explaining 61% of variance in the observed covariance matrix.

The TreeMix analysis with *m* = 1 migration edge (the best‐supported model based on log‐likelihood stabilization; Figure 6A) explained 61% of variance in the observed allele frequency covariance matrix (MSE = 0.000105). The log‐likelihood plateaued at *m* = 1 (80.45) and did not improve with additional edges, remaining constant for *m* = 1–3 and decreasing at *m* ≥ 4 (78.83). Therefore, *m* = 1 was selected as the most parsimonious model. The residual fit matrix indicated a generally good model fit, with all residuals < 0.002 and no systematic misfit between any population pair (Figure 6B). The resulting *m* = 1 model revealed two main genetic clusters: DICF + KGCF, and LICF + SICF (Figure 6C). A single migration event was inferred from the LICF/SICF ancestral lineage toward KGCF (migration weight = 0.336), suggesting approximately one‐third of KGCF ancestry derives from this lineage. DICF exhibited the shortest branch length (0.000195), indicating reduced genetic drift relative to other populations. Estimated effective migrants per generation (Nem) exceeded 1 for all population pairs (range: 11.3–47.8) (Figure S3). The highest gene flow was estimated between DICF and KGCF (Nem = 47.8), and the lowest between KGCF and LICF (Nem = 11.3) (Figure S3).

### 3.4 Isolation by Distance

Mantel tests revealed no significant correlation between geographic and genetic distances, indicating the absence of IBD. At the population level, the correlation was non‐significant based on *F*_ST_ values (*r* = −0.423, *p* = 0.750), and similarly, the individual‐based analysis showed no significant IBD pattern (*r* = −0.083, *p* = 0.935) (Figure S4).

### 3.5 Effective Population Size

Contemporary effective population size (Ne) was estimated for each population using the LD method (Waples 2006). At a minimum allele frequency threshold of Pcrit = 0.05, Ne estimates ranged from 86 (LICF) to 799 (KGCF), with DICF and SICF showing intermediate values of 170 and 104, respectively (Table S4). The LICF population had the smallest and most precisely estimated Ne (Ne = 86; 95% CI: 78–94). The SICF population also showed a relatively small Ne (Ne = 104; 95% CI: 56–152).

Ne estimates for KGCF were substantially larger (Ne = 799) but associated with wide confidence intervals (95% CI: 340–1257) (Figure S5A), likely reflecting reduced statistical power with 19 individuals. At lower Pcrit thresholds (0.02 and 0.01), KGCF estimates inflated to Ne > 5000 with confidence intervals spanning zero to infinity, indicating that the LD signal at rare allele frequencies is unreliable for this population size; the Pcrit = 0.05 estimate is therefore the most conservative and reportable value. DICF showed a moderate and well‐constrained Ne (~170 at Pcrit 0.05; ~212 at Pcrit 0.02). Sensitivity across Pcrit thresholds was greatest for KGCF and smallest for LICF and SICF, where estimates remained stable across all thresholds tested (Figure S5B).

## 4 Discussion

This study examined whether the brooding amphipod, *C. femoratus* , exhibits fine‐scale genetic connectivity in fragmented Antarctic habitats. Our findings revealed a weak population structure, indicating some genetic connectivity among populations. This is contrary to our initial hypothesis for *C. femoratus* , which predicted that a brooding life history and sedentary adult ecology would reduce dispersal potential and limit gene flow. The divergence of our results from expectations of high isolation suggests that *C. femoratus* shows genetic patterns consistent with relatively high connectivity for a brooding species, though this inference rests on a weak differentiation signal and should be interpreted cautiously. This was supported by low pairwise *F*_ST_ values and hierarchical variance decomposition showing that most genetic variation occurs within individuals (66.6%) and within populations (32.1% among individuals within populations), with only 1.3% attributable to differences among populations (*F*_ST_ = 0.013, *p* < 0.01), indicating weak differentiation and extensive shared genetic variation among populations. The Mantel test showed no significant IBD, indicating that geographic distance alone did not explain the observed pattern of genetic differentiation.

In marine environments, populations are often well‐connected, even over long distances (Manel et al. 2023). For the brooder *C. femoratus* , propagule dispersal is absent, so alternative mechanisms are required to explain population mixing. Rafting has been documented in the sub‐Antarctic, where amphipods were dispersed on buoyant Durvillaea kelp (Nikula et al. 2010). Although we did not identify specific rafting mechanisms, passive dispersal via floating substrates remains a plausible hypothesis given the region's complex oceanography with strong prevailing near‐shore currents. Floating algae or encrusted substrates may act as mobile habitats that can transfer individuals far from their origin (Fraser et al. 2022; López et al. 2018). In the Antarctic region, physical oceanographic processes, including the ACC, coastal eddies, and tidal mixing, may contribute to long‐distance transport among islands (Güller et al. 2020). The ecological flexibility of *C. femoratus* may increase the feasibility of this mechanism. Viable passive transport and survival on such substrates during transport may be possible in *C. femoratus* , which is an opportunistic omnivore that feeds on a variety of organic materials, including invertebrates, algae, and detritus (Núñez‐Pons et al. 2012; Seefeldt et al. 2017). This mechanism could help account for the unidirectional gene flow from SICF to KGCF revealed by the TreeMix, with a connection between the two populations ~200 km apart potentially facilitated by the prevailing BC that exists as a ca. 20 km wide current that flows in a north‐easterly direction along the southern coasts of the South Shetland Islands (Sangrà et al. 2017). Surface flow rates have been reported to range from 0.3 to 0.4 m s^−1^ (Sangrà et al. 2017), suggesting that passive transport over 200 km could theoretically be achieved within days. In addition to natural floating substrates, contemporary dispersal may also be facilitated by anthropogenic floating material associated with the numerous research stations and increasing maritime traffic in the region (Dawson et al. 2024). Although speculative, this possibility highlights the potential for human activities to unintentionally influence connectivity among Antarctic benthic species.

Positive *F*_IS_ values were observed across all populations, indicating a general heterozygote deficit. Although this pattern may be consistent with inbreeding, alternative explanations such as the Wahlund effect, null alleles, or the sampling of related individuals should also be considered. Effective population size estimates provide an indirect indication of the potential strength of genetic drift in each population and help place the within‐population diversity patterns described above into a demographic context, particularly for LICF. The LICF population had the smallest Ne (86; 95% CI: 78–94), suggesting stronger genetic drift in this population and providing a possible demographic explanation for its repeated identification as an outlier across independent analyses. PCA and admixture plots showed distinct clustering patterns for LICF, and although *K* = 2 was selected as the statistically optimal result based on cross‐entropy minimization, *K* = 3 further highlighted the relative differentiation of LICF. Because *K* = 2 may represent only the uppermost level of hierarchical structure even when additional substructure is present (Janes et al. 2017), we interpret the *K* = 3 pattern as exploratory support for finer‐scale differentiation in LICF. LICF also consistently returned the highest pairwise *F*_ST_ estimates against the other three sites and showed the strongest clustering tendency in the NJ tree. The SICF population also showed a small Ne (~104; 95% CI: 56–152), together with a high *F*_IS_ (0.55), low nucleotide diversity, and the highest Tajima's *D*. However, although the admixture analysis showed a somewhat distinct ancestry composition in SICF, this signal was not consistently supported by PCA or pairwise *F*_ST_, suggesting that SICF should not be interpreted as a clearly differentiated population cluster comparable to LICF. We note that SICF was the only population sampled by a baited trap at ~50 m depth, whereas the other populations were hand‐netted across intertidal regions. Depth and collection method are fully confounded for SICF, and their effects on its genetic metrics cannot be distinguished. Because *C. femoratus* broods offspring and releases them as non‐dispersing benthic juveniles, siblings likely remain clustered near the parent; a single trap deployment may therefore be more prone to capturing related individuals than multiple hand‐netting, potentially inflating *F*_IS_ and downwardly biasing the Ne estimate for SICF. We therefore cannot rule out that SICF's small Ne, in addition to its elevated *F*_IS_, partly reflects this sampling effect rather than a genuinely small breeding population. DICF showed a moderate and well‐constrained Ne (~170), consistent with its low *F*_IS_ and intermediate diversity levels. KGCF showed a larger but poorly constrained Ne estimate (799; 95% CI: 340–1257), suggesting a larger effective population but with insufficient statistical power to estimate it precisely at this sample size. Overall, Ne estimates should be interpreted cautiously, as LD‐based estimates can be sensitive to small sample sizes, related individuals, and allele‐frequency thresholds (Waples 2006; Waples and Do 2010; Waples and Anderson 2017). This uncertainty was particularly evident for KGCF, which showed a wide confidence interval and greater sensitivity to Pcrit.

While pairwise *F*_ST_ values remained low overall, their consistent statistical significance demonstrates the power of genome‐wide SNP data to detect small but biologically meaningful differences in allele frequencies among populations. Thus, the observed divergence of LICF should not be interpreted as strong isolation, but rather as evidence of fine‐scale population structuring further contextualized by demographic (low Ne) alongside allele‐frequency‐based evidence (*F*_ST_, PCA, admixture, and NJ tree) within an overall pattern of connectivity. This pattern suggests that LICF may be experiencing reduced gene flow or a distinct demographic history compared to the more genetically similar DICF, KGCF, and SICF populations. The repeated separation of LICF across analyses further suggests that ecological, oceanographic, or historical barriers may contribute to its divergence from the other island populations. For instance, small‐scale eddies associated with the BC (Sangrà et al. 2017), or persistent nearshore fast sea ice, could restrict the movement of rafting amphipods in and out of the LICF embayment where the population was sampled. A comparable pattern was reported in the Antarctic brooding microbivalve *K. subquadrata*, where Livingston Island populations exhibited strong local differentiation and unique haplotypes, likely associated with Bransfield Strait circulation and localized water retention that reduced dispersal and increased isolation (Levicoy et al. 2021). The TreeMix topology grouped LICF with SICF in a single cluster, whereas PCA and sNMF suggested LICF as the most genetically distinct population, separating from all others including SICF. This apparent inconsistency reflects the fundamentally different aspects of population history captured by each method. TreeMix models drift and covariance structure along a bifurcating tree, and therefore groups LICF with SICF because these two populations share a similar overall level of genetic drift relative to the root. In contrast, PCA and sNMF detect allele‐frequency differentiation more directly, as reflected in LICF's highest pairwise *F*_ST_ values (0.015–0.022). The two lines of evidence are therefore not contradictory but complementary: LICF may share common ancestry with SICF (as inferred by TreeMix) while having diverged most in allele frequencies from all populations (as detected by PCA and sNMF), likely reflecting population‐specific genetic drift potentially combined with restricted gene flow, since divergence from its ancestral lineage. We recommend that future studies with larger sample sizes and denser genomic coverage investigate whether this pattern reflects genuine isolation of the Livingston Island population or stochastic drift.

In addition to physical isolation, the divergence in the Livingston Island population could be linked to historical and contemporary environmental factors. Historically, Last Glacial Maximum (LGM)‐associated habitat contraction is known to reduce genetic diversity and foster differentiation, as reported for other Antarctic benthic species (Dambach et al. 2012; Díaz et al. 2018; Livingstone et al. 2016); however, our data include no explicit demographic dating analysis to test this scenario directly. Instead, our AMOVA analysis (1.3% among‐population variance) indicates weak overall genetic differentiation and estimated gene flow (Nem = 11.3–47.8 migrants per generation) exceeded the threshold generally considered sufficient to counteract population differentiation driven by genetic drift across all population pairs. Additionally, environmental parameters may play a significant role in shaping the genetic structure of amphipod populations (Chan et al. 2020; Xavier et al. 2023). Livingston Island has extensive glacial coverage and comparatively less ice‐free coastal habitat than King George or Deception Islands (Everett 1971; Hauck et al. 2007). This can result in habitat fragmentation and isolation, reducing opportunities for gene flow and enhancing genetic structuring. Although the specific mechanisms remain unclear, factors such as localized circulation, prolonged fast sea‐ice retention, or differences in substrate availability may limit connectivity with surrounding populations. Another key environmental factor is that Livingston Island generally experiences colder, more stable temperatures (Luengo‐S et al. 2025). These environmental differences in glacial extent, oceanographic conditions, and thermal stability represent plausible selective pressures that could contribute to the divergence of LICF. We propose them explicitly as hypotheses for future genotype‐environment association studies. No formal environmental association or selection‐outlier analysis was performed in the present study, and we do not claim to have demonstrated local adaptation.

The present study underscores the importance of localized processes in shaping genetic variation even across relatively small geographic scales in the Antarctic. The considerable connectivity observed among populations contrasts with the life history traits of *C. femoratus* , a brooding, direct‐developing amphipod with limited dispersal potential (Bregazzi 1972). Several studies have shown low gene flow and population structure in amphipods across geographical scales (Hogg et al. 2006; Weston et al. 2022). However, genetic connectivity in Antarctic shallow‐water amphipod species, such as the brooder *O. franklini*, has been reported at finer spatial scales, alongside strong structure between more distant regions, with a stepping‐stone dispersal mode inferred for this taxon (Baird et al. 2012). Our analyses revealed weak but statistically significant genetic structuring in *C. femoratus* , although extensive shared genetic variation was observed among populations. The evolutionary significance of this weak differentiation remains uncertain and requires further investigation. Future research should include ecological studies on differences in habitat‐associated macroalgae and food sources, as well as morphological analyses of key taxonomic characters, such as mouthpart structures. Moreover, sampling across different seasons and from more diverse locations within islands, as well as from other, more distant locations, will help minimize opportunistic sampling biases and strengthen the robustness of our results.

This study provides a valuable genetic baseline for tracking future shifts in population structure in response to ongoing environmental changes in the Antarctic coastal ecosystem. With regional warming, less persistent sea ice or changes in the availability and movement of rafting vectors may alter dispersal and connectivity among benthic communities on the South Shetland Islands. In addition, with human activities such as research station expansion and increasing maritime traffic continuing to intensify, long‐term monitoring of these populations will be essential to detect early signs of demographic or genetic erosion. Our findings suggest that even small‐scale environmental heterogeneity in the Antarctic may contribute to detectable genetic differentiation. As the Southern Ocean faces rapid environmental change, our results provide a foundation for assessing how fine‐scale differentiation and shifts in connectivity may influence the long‐term resilience and evolutionary trajectories of Antarctic benthic populations.

## Author Contributions

**Euna Jo:** data curation (equal), methodology (equal), resources (equal), validation (lead), visualization (equal), writing – original draft (equal). **Vahid Sepahvand:** conceptualization (equal), data curation (equal), formal analysis (lead), methodology (equal), visualization (equal), writing – original draft (equal). **Yong‐Uk Ahn:** investigation (equal), methodology (equal), resources (equal), writing – review and editing (equal). **Seunghyun Kang:** writing – review and editing (equal). **Miles Lamare:** writing – review and editing (equal). **Ceridwen Fraser:** writing – review and editing (equal). **Jee‐Hoon Kim:** writing – review and editing (equal). **Sanghee Kim:** conceptualization (equal), project administration (lead), writing – review and editing (equal).

## Funding

This work was supported by the Korea Polar Research Institute (KOPRI) grant funded by the Ministry of Oceans and Fisheries (KOPRI PE26140) and the management of the Marine and Fishery Bio‐resources Center (2026) funded by the National Marine Biodiversity Institute of Korea (MABIK). Euna Jo was supported by the National Research Foundation of Korea (NRF) grant funded by the Korean government (MSIT) (RS‐2026‐25492996). Vahid Sepahvand was supported by the Antarctic Science Platform, funded by the MBIE New Zealand Antarctic Science Platform (ANTA‐1801) and the University of Otago.

## Conflicts of Interest

The authors declare no conflicts of interest.

## Supporting information


**Figure S1:** Cross‐entropy plot from sNMF ancestry analysis of *Cheirimedon femoratus* populations.
**Figure S2:** Neighbor‐joining (NJ) phylogenetic tree of 87 *Cheirimedon femoratus* individuals from four populations in the South Shetland Islands, constructed from 8837 genome‐wide SNPs using Prevosti's genetic distance.
**Figure S3:** Gene flow among *Cheirimedon femoratus* populations, estimated as the effective number of migrants per generation (Nem) using Wright's (1951) island model approximation: Nem = (1 − *F*_ST_)/(4*F*_ST_).
**Figure S4:** (A) Mantel test showing no significant correlation between geographic and genetic distances among four populations of *Cheirimedon femoratus*. (B) Mantel test based on the individuals showing no significant correlation between geographic and genetic distances among four populations of *C. femoratus* .
**Figure S5:** Effective population size (Ne) for four *Cheirimedon femoratus* populations using the linkage disequilibrium (LD) method (Waples 2006).
**Table S1:** Per‐population summary statistics: inbreeding coefficient (*F*_IS_), Tajima's *D*, and percentage of genomic windows with significant Tajima's *D* (*p* < 0.05).
**Table S2:** Full analysis of molecular variance (AMOVA) results based on the Weir and Cockerham (1984) *F*_ST_ estimator implemented in hierfstat v0.5‐11.
**Table S3:** Pairwise *F*_ST_ values (Weir and Cockerham 1984) among four *Cheirimedon femoratus* populations, with 95% confidence intervals from bootstrap resampling (999 replicates).
**Table S4:** Effective population size (Ne) estimates for four *Cheirimedon femoratus* populations using the linkage disequilibrium (LD) method (Waples 2006).

## Data Availability

The raw demultiplexed data have been deposited on the NCBI Sequence Read Archive under BioProject accession no. PRJNA1454734 (https://www.ncbi.nlm.nih.gov/sra). All analysis scripts and processed data files have been deposited on Zenodo (https://zenodo.org/records/21764994; DOI: 10.5281/zenodo.21764994).
