# Supplementary material for: Fine‐Scale Population Genomics Reveals Genetic Differentiation in the Brooding Amphipod Cheirimedon femoratus Across the South Shetland Islands, Antarctica
Source: Ecol Evol. 2026 Sep 8;16(9):e74336. doi: 10.1002/ece3.74336 (PMC13553554; doi:10.1002/ece3.74336)
Supplement: Supplementary file 1 [file ECE3-16-e74336-s001.docx]

**Supplementary Materials**

Supplementary Figure S1. Cross-entropy plot from sNMF ancestry analysis of *Cheirimedon femoratus* populations.

Supplementary Figure S2. Neighbor-joining (NJ) phylogenetic tree of 87 *Cheirimedon femoratus* individuals from four populations in the South Shetland Islands, constructed from 8,837 genome-wide SNPs using Prevosti's genetic distance. The tree is midpoint-rooted. Tip points are color-coded by sampling location. Bootstrap support values (100 replicates) are shown for nodes with >= 70% support.

Supplementary Figure S3. Gene flow among *Cheirimedon femoratus* populations, estimated as the effective number of migrants per generation (Nem) using Wright's (1951) island model approximation: Nem = (1 − F_ST_) / (4F_ST_). Line width is proportional to Nem. All population pairs show Nem > 1 (range: 11.3-47.8).

Supplementary Figure S4. (A) Mantel test showing no significant correlation between geographic and genetic distances among four populations of *Cheirimedon femoratus*. (B) Mantel test based on the individuals showing no significant correlation between geographic and genetic distances among four populations of *C. femoratus.*

Supplementary Figure S5. Effective population size (Ne) for four *Cheirimedon femoratus* populations using the linkage disequilibrium (LD) method (Waples, 2006). Ne and 95% jackknife confidence intervals shown for Pcrit = 0.05 and 0.02. n = number of individuals used in analysis. (A) Points estimates +95 jackknife CI at Pcrit =0.05. (B) Sensitivity to Pcrit threshold (log scale).

Supplementary Table S1. Per-population summary statistics: inbreeding coefficient (F_IS_), Tajima's D, and percentage of genomic windows with significant Tajima's D (p < 0.05).

| **Population** | **F_IS_** | **Median Tajima's D** | **% windows p<0.05** |
| --- | --- | --- | --- |
| DICF | 0.207 | 0.198 | 4.6% |
| KGCF | 0.204 | 0.206 | 3.9% |
| LICF | 0.319 | 0.229 | 4.6% |
| SICF | 0.558 | 0.411 | 4.8% |

Supplementary Table S2. Full Analysis of Molecular Variance (AMOVA) results based on the Weir and Cockerham (1984) F_ST_ estimator implemented in hierfstat v0.5-11. Significance assessed by permutation (999 replicates).

| **Source** | **df** | **Variance component** | **% Variation** | **Statistic** |
| --- | --- | --- | --- | --- |
| Among populations | 3 | 29.705 | 1.3% | F_ST_ = 0.013, p < 0.01 |
| Among individuals within populations | 83 | 735.249 | 32.1% | F_IS_ = 0.325 |
| Within individuals | 87 | 1528.645 | 66.6% | F_IT_ = 0.334 |
| Total | 173 | 2293.598 | 100% | — |

Supplementary Table S3. Pairwise F_ST_ values (Weir and Cockerham 1984) among four *Cheirimedon femoratus* populations, with 95% confidence intervals from bootstrap resampling (999 replicates). All comparisons were statistically significant.

| **Population pair** | **Pairwise F_ST_** | **95% CI lower** | **95% CI upper** | **p-value** |
| --- | --- | --- | --- | --- |
| DICF - KGCF | 0.0052 | 0.0040 | 0.0064 | < 0.001 |
| DICF - LICF | 0.0153 | 0.0136 | 0.0169 | < 0.001 |
| DICF - SICF | 0.0081 | 0.0063 | 0.0100 | < 0.001 |
| KGCF - LICF | 0.0216 | 0.0195 | 0.0236 | < 0.001 |
| KGCF - SICF | 0.0096 | 0.0078 | 0.0116 | < 0.001 |
| LICF - SICF | 0.0199 | 0.0176 | 0.0226 | < 0.001 |

Supplementary Table S4. Effective population size (Ne) estimates for four *Cheirimedon femoratus* populations using the linkage disequilibrium (LD) method (Waples, 2006). Ne and 95% jackknife confidence intervals shown for Pcrit = 0.05 and 0.02. N = number of individuals used in analysis.

| **Population** | **N** | **Ne (Pcrit 0.05)** | **95% CI** | **Ne (Pcrit 0.02)** | **95% CI** | **Reliability** |
| --- | --- | --- | --- | --- | --- | --- |
| DICF | 22 | 169.8 | 151.9–187.8 | 212.3 | 185.8–238.8 | Reliable |
| KGCF | 19 | 798.7 | 340.1–1257.4 | 5545.1 | 0–31112.6 | Wide CI — use Pcrit 0.05 only |
| LICF | 16 | 86.0 | 78.1–93.9 | 106.8 | 95.0–118.5 | Reliable — smallest Ne |
| SICF | 19 | 103.8 | 56.0–151.6 | 103.8 | 56.0–151.6 | Acceptable |
